# Comprehensive analysis of published studies involving systemic treatment for chondrosarcoma of bone between 2000 and 2013

**DOI:** 10.1186/2045-3329-4-11

**Published:** 2014-08-12

**Authors:** Annemiek M van Maldegem, Judith VMG Bovée, Hans Gelderblom

**Affiliations:** 1 Department of Clinical Oncology, Leiden University Medical Centre, Albinusdreef 2, 2333 ZA Leiden, The Netherlands; 2 Department of Pathology, Leiden University Medical Centre, Leiden, The Netherlands

**Keywords:** Chondrosarcoma, Systemic treatment, Clinical trial

## Abstract

**Background:**

The majority of patients with chondrosarcoma of bone have an excellent overall survival after local therapy. However, in case of unresectable locally advanced or metastatic disease the outcome is poor and limited treatment options exist. Therefore we conducted a survey of clinical phase I or II trials and retrospective studies that described systemic therapy for chondrosarcoma patients.

**Materials and methods:**

Using PubMed, clinicaltrials.gov, the Cochrane controlled trial register and American Society of Clinical Oncology (ASCO) abstracts a literature survey was conducted. From the identified items, data were collected by a systematic analysis. We limited our search to semi-recent studies published between 2000 and 2013 to include modern drugs, imaging techniques and disease evaluations.

**Results:**

A total of 31 studies were found which met the criteria: 9 phase I trials, 11 phase II and 8 retrospective studies. In these studies 855 chondrosarcoma patients were reported. The tested drugs were mostly non-cytotoxic, either alone or in combination with another non-cytotoxic agent or chemotherapy. Currently two phase I trials, one phase IB/II trial and three phase II trials are enrolling chondrosarcoma patients.

**Conclusion:**

Because chondrosarcoma of bone is an orphan disease it is difficult to conduct clinical trials. The meagre outcome data for locally advanced or metastatic patients indicate that new treatment options are needed. For the phase I trials it is difficult to draw conclusions because of the low numbers of chondrosarcoma patients enrolled, and at different dose levels. Some phase II trials show promising results which support further research. Retrospective studies are encouraged as they could add to the limited data available. Efforts to increase the number of studies for this orphan disease are urgently needed.

## Background

Chondrosarcoma (CS) is the second most common primary bone sarcoma in humans, but with an estimated incidence of 0.2 in 100.000 patients per year it is still a very rare disease [1]. CS mostly affects adults between the age of 20 and 60 [2]. CS belong to a very diverse group of tumors having in common the production of cartilaginous matrix. Almost 90% of the CS are of the conventional subtype, but there are also the more rare subtypes with their own distinct histological and clinical features including clear cell, mesenchymal and dedifferentiated CS [3]. The prognosis for patients with CS is very diverse with a very good prognosis for atypical cartilaginous tumour/CS grade I which are slow growing and do not metastasize and a poor prognosis for grade III CS which have a high risk, up to 70%, for local recurrence and metastasis [4,5]. Currently the most commonly used treatment option for atypical cartilaginous tumour/CS grade I is curettage with local adjuvants which is usually enough to cure the patient. However, for grade II and grade III CS en bloc resection is required. If a patient has unresectable or metastasized disease the current treatment options are very limited. CS has always been considered to be chemotherapy and radiotherapy resistant and it was assumed that patients would not benefit from non-surgical treatment. However, new preclinical work and retrospective studies show that there may be a place for non-cytotoxic, chemo- and radiotherapy in the treatment of CS [6,7]. In the last decades more knowledge has become available about the molecular background of the different CS subtypes (for review see [8-10]). Investigators have been trying to find new systemic treatment options for these patients through phase I and II clinical trials (no phase III studies were ever conducted). Because CS is such a rare disease, and high grade metastatic or unresectable disease is even more uncommon, the number of patients in these trials is however low and thereby it is difficult to give a clear answer to the question whether a new drug improves outcome or not. Here we report an overview of a survey we conducted on published and presented phase I and II clinical trials and retrospective studies on systemic therapy enrolling CS patients, published from 2000 until 2013. We also include the studies that are enrolling patients at this moment.

## Material and methods

### Search strategy

To collect phase I and II and retrospective studies which included CS patients we used the search machines PubMed, clinicaltrials.gov, the Cochrane controlled trial register and American Society of Clinical Oncology (ASCO) abstracts. For the search criteria we used the terms [chondrosarcoma] AND [phase I OR phase II OR retrospective] AND [clinical trial]. To check for missed articles we widened the search to [sarcoma] AND [phase I OR phase II] AND [clinical trial] and compared the results. When multiple reports from the same trial were published we only used the article with the longest follow up time. Publications were used if they 1) described results from an early phase clinical trial in which CS patients were included, either prospective or retrospective, 2) were written in English. The latest search was performed in December 2013. Studies on extraskeletal myxoid CS were excluded.

Data were collected from trials published between 2000 and 2013 to include modern drugs, imaging techniques and disease evaluations. Data extraction was done by one of the authors (A.v.M) and a systemic analysis was applied that is normally used for meta-analysis [11]. From all reports author name, year of publication, number of patients, intervention and outcome data were noted.

## Results

**Figure 1:**
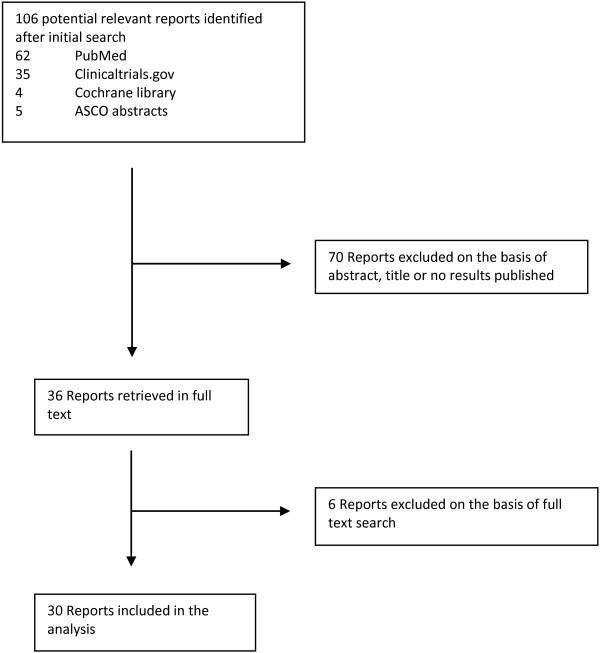
Flow-chart showing the search method to identify the relevant clinical reports for our analysis.

**Figure 2:**
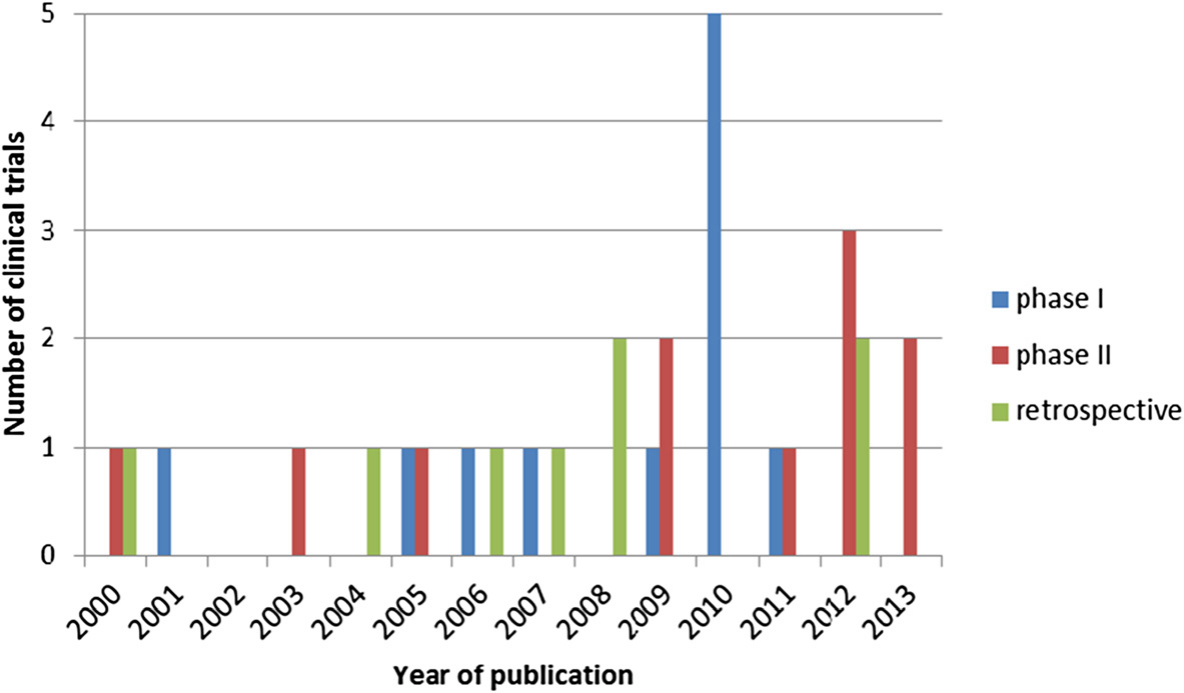
Overview of the number of clinical trials included in the analysis according to the year of publication with phase I trials in blue, phase II trials in red and the retrospective trials in green.

From 2000 until 2013 a total of 31 phase I, II or retrospective clinical trials were reported that enrolled 1 or more CS patients: 11 phase I, 11 phase II and 8 retrospective studies. Figure 1 shows a flow-chart indicating the search method for clinical trials included in this analysis. Figure 2 shows a timetable with the number and type of clinical trials that met our criteria and their time of publication showing an increasing number of publications from 2004 onwards. The data from the trials that were included in this study are shown in Table 1 for the phase I trials, Table 2 for phase II trials and Table 3 for the results of the retrospective studies. In the clinical trials that were identified a total of 1927 patients were included of which 855 are patients with CS. Histological subtypes included were conventional, dedifferentiated and mesenchymal. The actual number of patients with CS may be higher because in some of the phase I trials only the CS patients who had an objective response or stable disease were reported but more may have been enrolled. The drugs that were being tested were mostly non-cytotoxic in the phase I trials, either alone or in combination with another non-cytotoxic agent or conventional chemotherapy in the phase II trials. For the retrospective studies all treatments were conventional chemotherapy based. In the phase I trials of the 13 included CS patients there were no complete response (CR), 2 (15%) partial response (PR) and 7 (54%) stable disease (SD). For the phase II trials 156 CS patients were enrolled with 2 (1.3%) CR, 2 (1.3%) PR and 21 (13.4%) SD. The results on the clinicaltrials.gov website for current trials are shown in Table 4. Two phase I trials, one phase IB/II trial and three phase II trials were found. There are no phase III trials that are currently or were ever recruiting CS patients.

**Table 1:** Phase I trials included in the analysis showing the name of the author, year of publication, number of patients enrolled in the trial, the intervention that was tested and the outcome

**Author**	**Year**	**No of patients**	**Intervention**	**Outcome for CS patients**
Serrone et al. [12]	2001	44 advanced sarcoma (2 CS)	Group A: IFOS short infusion + EPI	Not mentioned
			Group B: IFOS continuous infusion + EPI	
Schwartz et al. [13]	2005	38 advanced solid tumors with 6 sarcoma with at least 1 CS	LY293111 (Leucotriene-B4 receptor antagonist)	1 SD
Levine et al. [14]	2006	51 advanced malignancies with 7 sarcoma with at least 1 CS	Veglin (VEGF antisense oligodeoxynucleotide)	1 SD
Lockhart et al. [15]	2007	18 advanced malignancies (1 CS)	MAC-321 (analogue of docetaxel)	1 SD
Chawla et al. [16]	2009	20 advanced sarcoma (1 CS)	Rexin-G (tumor-targeted retrovector)	Not mentioned
			Group A: 2 times a week	
			Group B: 3 times a week	
Camidge et al. [17]	2010	50 advanced malignancies with 11 sarcoma with at least 1 CS	PRO95780 (death receptor 5 antibody)	1 SD
Herbst et al. [18]	2010	71 advanced malignancies with 9 sarcoma with at least 2 CS	rhApo2L/TRAIL (death receptor activator)	2 PR
			Cohort 1: dose escalation	
			Cohort 2: treated at MTD	
Olmos et al. [19]	2010	29 advanced sarcoma (1 CS)	Figitumumab (IGF1R antibody)	1 SD
Qeuk et al. [20]	2010	21 advanced solid tumors (1 CS)	Everolimus (mTOR inhibitor) and figitumumab (IGF1R antibody)	1 SD
Stroppa et al. [21]	2010	8 advanced sarcoma (1 CS)	Doxorubicin + IFOS	Not mentioned
Pacey et al. [22]	2011	25 advanced solid tumors with at least 1 CS	Alvespimycin (heat shock protein 90 inhibitor)	1 SD

*CS* chondrosarcoma, *EPI* epirubicin, *IFOS* ifosfamide, *PR* partial response, *SD* stable disease.

**Table 2:** Phase II trials included in the analysis with the name of the author, year of publication, number of patients enrolled in the trial, the intervention that was tested and the outcome

**Author**	**Year**	**No of patients**	**Intervention**	**Outcome for CS patients**
Merimsky et al. [23]	2000	18 advanced sarcoma (3 CS)	Gemcitabine	2 SD
Skubitz et al. [24]	2003	47 advanced sarcoma (1 CS)	Pegylated-liposomal doxorubicin	Not mentioned
Nooij et al. [25]	2005	37 bone sarcoma (16 CS)	Doxorubicin + cisplatin	2 CR
		Group A: operable, non-metastatic (4 CS)		3 SD
		Group B: inoperable, metastatic (12 CS)		
Maki et al. [26]	2009	145 advanced sarcoma (2 CS)	Sorafenib (multi-tyrosine kinase inhibitor)	Not mentioned
Pacey et al. [27]	2009	26 advanced sarcoma (2 CS)	Sorafenib(multi-tyrosine kinase inhibitor)	1 SD
Grignani et al. [28]	2011	26 advanced chondrosarcoma	Imatinib mesylate (c-kit/PDGFR inhibitor)	8 SD
				OS 11 months
Fox et al. [29]	2012	53 advanced sarcoma (25 CS)	Gemcitabine + docetaxel	2 PR
Italiano et al. [30]	2012	40 advanced CS	GDC-0449/vismodegib (hedgehog inhibitor)	4 SD (of first 17 patients)
Schuetze et al. [31]	2012	49 advanced sarcoma (2 CS)	Sirolimus (mTOR inhibitor) + cyclophosphamide	1 SD
Ha et al. [32]	2013	36 advanced sarcoma	Cetuximab (EGFR antibody)	PD
		Group A: EGFR+		
		Group B: EGFR- (1 CS)		
Schwartz et al. [33]	2013	388 advanced sarcoma	Cixutumumab (IGF1R antibody) + temsirolimus (mTOR-inhibitor)	Not mentioned
		Group A: IGF-1R + soft tissue sarcoma		
		Group B: IGF-1R + bone sarcoma (20 CS)		
		Group C: IGF-1R- sarcoma (18 CS)		

*CR* complete response, *CS* chondrosarcoma, *OS* overall survival, *PD* progressive disease, *PFS* progression free survival, *PR* partial response, *SD* stable disease.

**Table 3:** Retrospective trials included in the analysis showing the name of the author, year of publication, number of patients enrolled in the trial, the intervention that was tested and the outcome

**First author**	**Year of publication**	**No of patients**	**Intervention and number of patients**	**Outcome of systemic therapy**
Mitchell [34]	2000	22 resectable dediff CS	Doxorubicin based chemotherapy + surgery	1/5 > 90% necrosis after preoperative chemo
			5 preoperative chemotherapy and 6 postoperative, 11 no chemotherapy	5 year OS with chemotherapy 36%, without 0%
Dickey [35]	2004	42 local or advanced dediff CS	15 Surgery	5 year OS and median OS: with surgery 11.8% and 6.4 months
			22 surgery and chemotherapy 5 other	
				With surgery and chemotherapy 4% and 8.4 months
Staals [36]	2006	102 local or advanced dediff central CS	68 Surgery	Median survival 18 vs 23 months (not significant)
			25 surgery + chemotherapy	
			9 palliative care	
Cesari [7]	2007	24 local or advanced mesenchymal CS	24 surgery of which:	10 year OS:
			5 + RT	Surgical remission 27%
			12 + chemotherapy	Non-surgical remission 0%
				Complete surgical remission 10 year DFS:
				With chemotherapy 76%, without 17%
Grimer [37]	2008	266 local and 71 advanced dediff CS	207 Surgery	Local disease
			90 chemotherapy	5 year OS:
			40 surgery + chemotherapy	With chemotherapy 33%, without 25% (p = 0.11)
				Advanced disease
				Median OS: With chemotherapy 7 months, without 3 months (not significant)
Dantonello [38]	2008	14 local mesenchymal CS	12 Surgery + chemotherapy	10 year OS 64%
			2 surgery + RT	
Bernstein-Molho [39]	2012	9 advanced CS	Sirolimus + cyclophosphamide	OR 11%
				SD 56%
				PFS 15 months
Italiano [5]	2012	98 advanced CS	Doxorubicin based chemotherapy	OR 14%
				SD 31%
				PFS 5.3 months
				OS 19 months

*CS* chondrosarcoma, *Dediff* dedifferentiated, *DFS* disease free survival, *OR* objective response, *OS* overall survival, *PFS* progression free survival, *RT* radiotherapy, *SD* stable disease.

**Table 4:** Overview of the trials currently recruiting chondrosarcoma patients, showing the clinicaltrials.gov number, title of the study and the phase

**Clinicaltrials.gov number**	**Title**	**Phase**
NCT01522820	Vaccine therapy with or without sirolimus in treating patients with NY-ESO-1 expressing solid tumors	I
NCT01643278	Dasatinib and ipilimumab in treating patients with gastrointestinal stromal tumors or other sarcomas that cannot be removed by surgery or are metastatic	I
NCT01154452	Vismodegib and gamma-secretase/notch signalling pathway inhibitor RO4929097 in treating patients with advanced or metastatic sarcoma	IB/II
NCT01330966	Study of pazopanib in the treatment of surgically unresectable or metastatic chondrosarcoma	II
NCT00928525	Imatinib in patients with desmoid tumor and chondrosarcoma	II
NCT01653028	Alisertib in treating patients with advanced or metastatic sarcoma	II
Not yet assigned	A phase 2, single arm, multi center trial evaluating the efficacy of the combination of sirolimus and cyclophosphamide in metastatic or unresectable myxoid liposarcoma and chondrosarcoma.	II

## Discussion

CS is a primary bone cancer that in most patients can be cured by local treatment alone. When tumors are unresectable, either because of locally advanced or metastatic disease, systemic treatment options are very limited due to the current view that non-surgical treatment options have no benefit. Currently for advanced CS patients the outcome is poor with an overall survival of less than two years [5,40,41].

The general insensitivity to chemotherapy in CS may be due to activation of anti-apoptotic and pro-survival pathways and therefore future treatment of advanced CS patients could benefit from targeted agents that specifically interfere with these pathways, rendering the tumours more sensitive to the conventional chemotherapeutic agents [8,9,40]. For instance, the anti-apoptotic proteins Bcl-2 and Bcl-XL are highly expressed in all CS subtypes and the BH-3 mimetic ABT-737 renders CS cell lines sensitive to the conventional chemotherapeutic agents doxorubicin and cisplatin [10,40]. Survivin, a member of the inhibitor of apoptosis protein family, is expressed in CS samples and RNA interference targeted on survivin results in cell cycle arrest and increased apoptotic rates in CS cell lines [42]. However, despite promising results in vitro, some novel approaches never make it to a clinical trial. An example of this is the combination treatment of the Bcl-2 inhibitor ABT-737 and doxorubicin. Drug companies were not interested to supply drug for a clinical trial so it remains unclear if this combination is beneficial to patient outcome.

Two recent retrospective studies as well as animal studies suggested that a subgroup of the patients may benefit from non-cytotoxic agents, chemotherapy, radiotherapy or a combination [6,7,41]. In systemic treatment, being either a doxorubicin-containing chemotherapy regimen or non-cytotoxic drugs as imatinib and sirolimus, significantly improved survival compared to no treatment in central CS patients with unresectable disease was shown [41]. For patients with only locally advanced disease radiotherapy may be a good therapeutic option with a significant survival benefit compared to no treatment. Patients with mesenchymal or dedifferentiated CS may also benefit from systemic treatment [6], and further clinical studies are warranted looking at specific CS subtypes.

Recently also new treatment options were tested in clinical trials such as the hedgehog (Hh) inhibitor IPI-926. Hedgehog signaling was previously shown to be important in CS genesis [43-45]. CS xenograft models were treated with IPI-926 and showed a downregulation of the Hh pathway in the tumors and a significant growth inhibition in both newly planted as well as established CS tumours with a mean of 43% [30]. Because of the strong preclinical results a randomized phase II trial studying the effect of IPI-926 compared to placebo in metastatic or locally advanced CS patients was conducted (NCT01310816). The study showed that IPI-926 is well tolerated but there was no difference in PFS or OS compared to placebo. However a small subset of patients had minor reductions in tumour size. Another Hh inhibitor that was tested in a phase II trial including advanced CS patients was GDC-0449, also called vismodegib (NCT01267955). This study did not meet its primary endpoint, but the results suggested activity of the drug in a subset of patients with progressive grade 1 or 2 conventional CS [46]. Despite the fact that in both studies only a small subset showed benefit, it is important to do these trials even if the results do not seem promising for the whole patient group. By studying the tumor tissue of the small subset of responders, we may learn to better understand the mode of action. Moreover, this will enable the identification of biomarkers to predict which patients do respond to the treatment to improve patient selection in future trials.

According to the clinicaltrials.gov website currently 6 clinical phase I or II trials are enrolling CS patients, in all of these trials non-cytotoxic agents are being tested either alone or in combination. These treatments are based on preclinical work that has been conducted over the last years and which shows promising results. An example of this is the mTOR pathway. Dysregulation of mTOR signaling can be found in many tumor types, however in clinical trials inhibitors of mTOR so far show only modest results, which may be due to activation of the PI3K/Akt pathway. In CS, both mTOR and AKT were shown to be activated [28,42]. Dual inhibition using BEZ235 dramatically decreased growth of CS cell lines and xenografts [28]. The euroSARC consortium, http://www.eurosarc.eu, is starting a phase II study in unresectable conventional, dedifferentiated and mesenchymal CS with the combination of sirolimus and cyclofosfamide. An exploratory analysis of the mTOR pathway is foreseen in this study with pharmacokinetic assays on tumour biopsies taken before and after treatment (http://www.eurosarc.eu).

We conducted a search for all clinical phase I or II trials and retrospective studies that included CS patients. Because we intended to include modern imaging and study designs the survey was limited to the period from 2000 until 2013. The list of the phase I trials that met our search criteria is probably not completely reflecting the actual number of CS patients enrolled in phase I clinical trials, which is caused by the search strategy by which trials that included CS patients without mentioning them in the abstracts are very difficult to find. Some of the phase I trials could be found by using the Cochrane Controlled Trial Register or the clinicaltrials.gov database, although not all trials are registered in these databases. In the phase I trials, with an average of 34 recruited patients, only a small number of CS patients were enrolled at various dose levels which make it difficult to conclude on the effect of these new treatment options specifically for CS patients. For the phase II trials sarcoma patients were enrolled in different strata. Some strata included CS patients while the studies by Grignani and Italiano were restricted to CS patients only [46,47].

For the retrospective studies it was difficult to make definite conclusions on the effect of systemic therapy because many different chemotherapy treatment regimens were used in patients with different stages of disease and different CS subtypes. As the biological behavior of the subtypes differs much with therefore expected different outcomes it is hard to conclude from studies where the specific subtype is not reported. The study by Cesari [48] is very interesting because it shows that mesenchymal CS patients who had a complete surgical remission may benefit from adjuvant chemotherapy with a 10 year disease free survival that improves from 10% without chemotherapy to 76% with chemotherapy. This is in line with previous studies and the current opinion is that mesenchymal CS is a chemotherapy sensitive tumor and that patients therefore benefit from chemotherapy treatment [25,29]. For the other CS subtypes it has generally been thought that they are insensitive to conventional chemotherapy, although for dedifferentiated CS activity was shown in individual cases and it is still undefined if chemotherapy treatment is effective [3,12,49]. A retrospective study with 337 dedifferentiated CS patients shows that the prognosis remains dismal, however an improvement of survival, not significant, in patients receiving chemotherapy who are under 60 years of age and had limb salvage treatment was found [13]. Also for conventional CS more evidence is currently found that systemic treatment does improve survival [41].

In conclusion, CS is a difficult patient population to study in clinical trials. It is a very rare disease and most patients can be cured by surgery alone, which makes the group of patients that might need adjuvant or palliative treatment even smaller. This is also one of the reasons why it is very difficult to receive funding for these studies. However, from the current poor prognosis of non-resectable locally advanced or metastatic CS patients it is very clear that there is an unmet medical need and new treatment options are warranted. To improve the number of new treatment options for these patients it is essential to collaborate and share data on research so that future clinical trials have a sound biological rationale and can be conducted with as few patients as possible in a short timeframe.

## Competing interests

The authors declare that they have no competing interests.

## Authors’ contributions

HG conceived the study. AVM collected data. AVM and HG wrote the paper and JB made comments. All authors read and approved the final manuscript.
